# Factors driving the biomass and species richness of desert plants in northern Xinjiang China

**DOI:** 10.1371/journal.pone.0271575

**Published:** 2022-07-22

**Authors:** Cheng Fei, Yi Qiang Dong, Sha Zhou An

**Affiliations:** College of Grassland Science XinJiang Agricultural University, Urumchi, The Xinjiang Uygur Autonomous Region, China; Feroze Gandhi Degree College, INDIA

## Abstract

The desert ecosystem is an important part of the terrestrial ecosystem. Accurate estimations of the biomass and species richness of desert plants are of great value for maintaining ecosystem stability; however, current assessments remain a challenge due to the large spatial heterogeneity in biomass and species richness and difficulties posed by time-consuming field surveys, particularly in remote areas. In the present study, There were 527 sampling sites, and each sampling site contained approximately 9 quadrats. Approximately 4500 quadrats in total were taken from the Junggar Desert of northern Xinjiang, and the spatial distribution and factors driving the biomass and species richness of the desert ecosystem were quantitatively analyzed. The results showed that the average aboveground biomass, belowground biomass, litter, and the Patrick index of the Junggar Desert in northern Xinjiang were 115.42 gm−2, 924.77 gm−2, 13.06 gm−2, and 63, with values ranging from 2–708.12 gm−2, 120.25–3537.3 gm−2, 2–56.46 gm−2, and 0–377, respectively, The mean of the variation coefficient was 56.19%, 41.16%, 62.16% and 73.83%, suggesting moderate variation. The result is affected by the differences between the desert environment and climate. Climate factors had a relatively large impact on species richness, and the variation coefficient of species richness was large, indicating a large degree of dispersion of species richness. The direct influence of environmental and climatic factors on underground biomass (BGB) is relatively small, and its coefficient of variation is small. The spatial distribution of biomass and species richness in northern Xinjiang gradually decreased from west to east. Redundancy analysis showed that climate was the main factor driving desert biomass and species richness in northern Xinjiang, with an average independent explanatory power of 20.38% and 18.57%, respectively. Structural equation modeling indicated that climate factors, elevation, and community coverage had a direct positive effect on the aboveground biomass of the desert plants in northern Xinjiang and a direct negative effect on the belowground biomass. Moreover, climate factors and biological factors showed a direct positive effect on the species richness in northern Xinjiang.

## Introduction

Biomass represents the ability of an ecosystem to obtain energy, which is one of the most fundamental quantitative characteristics for understanding community structure and function [1]. Biodiversity research is a pertinent topic in global ecosystem research [2, 3]. One of the most important components of ecological research is species diversity [4]. The better the environmental conditions, the greater the species richness, and the more stable the ecosystem [2, 5, 6]. CO_2_ in the atmosphere is fixed in the ecosystem in the form of carbohydrates through photosynthesis, which plays an important role in regulating the global carbon cycle, climate, biodiversity, and ecological balance [7–11]. Therefore, it is of great significance to accurately estimate the biomass and species richness of desert communities so as to maintain ecosystem stability. The majority of current studies on biomass, especially belowground biomass, have focused on forests, grasslands, and arable land [12–14], whereas few studies have evaluated the biomass of deserts, which typically have sparse vegetation. Although there have been numerous studies on the species diversity of deserts and grasslands [15–19], most have focused on a single community type and on the species diversity of grasslands in Northeast China and Inner Mongolia. Therefore, an in-depth analysis of the variation characteristics of biomass and species richness in desert areas as well as the factors driving their spatial distribution is required.

The arid desert area is a typical ecologically fragile area, and the complexity of the environment results in great differences in productivity and species composition [20]. Some studies have pointed out that the contribution of desert ecosystems to terrestrial ecosystems is almost negligible [21]. However, others believe that despite the low vegetation coverage, the desert vegetation is widely distributed, and its potential for carbon storage in mitigating global climate change cannot be ignored [22, 23]. Desert ecosystems may be the “carbon sinks” that the global carbon cycle has been seeking. Biomass is an important carbon carrier in ecosystem cycles [24, 25]. Currently, the average biomass of global desert areas is merely about 7 Mg/hm^2^, yet increasing attention has been placed on the role deserts play in the global carbon cycle [26]. The Sonoran Desert in North America accounts for 1% of the global desert area, and its biomass accounts for 4.4% of the global biomass, showing strong potential to become a carbon sink zone [24, 27]. Thus, it has also attracted widespread academic attention [2, 28, 29]. Xinjiang in northwestern China is a large area of desert that was formed in the Tertiary Age. It is part of the Asian-African desert area and accounts for more than 42% of the total land area of Xinjiang. The results will be of great theoretical and practical significance for the rational development of grasslands, desert conservation, and climate change mitigation.

Biomass is the total amount of living organisms or total energy stored in a unit surface area during an observation period and is also referred to as the standing crop [2]. Species richness is the number of species in a unit area in a certain plant community. The better the environmental conditions, the greater the species richness [2]. Belowground biomass (BGB) is an important component of carbon accumulation in vegetation since the biomass is mainly distributed belowground [30, 31]. In contrast, aboveground biomass (AGB) is an important means by which grassland ecosystems obtain energy and fix CO_2_ [32]. Due to the lack of a simple and efficient method for BGB measurement, the progress on BGB has long been slower than that of AGB [30, 31]. According to existing studies, climate factors (e.g., water and heat) and soil moisture content have a large impact on grassland biomass [31]. Deserts occupy a substantial portion of the global terrestrial ecosystem and thus more attention should be placed on the desert ecological environment. The climatic conditions in different desert areas differ, which results in certain differences in the types and biomass characteristics of different deserts [33]. There is a strong correlation between vegetation diversity and environmental factors [33, 34]. A survey of 195 plant communities in the desert area of Northwest China supported the water-energy dynamics hypothesis, which suggests that the soil water content, precipitation, and spatial factors are the main factors limiting species diversity. The total explanatory power of the three reached 48.08% [35]. Another study by also showed that changes in plant diversity were affected by factors such as paleo climate, modern climate, elevation, soil nutrients, and community biomass [36].

Both the energy hypothesis and the environmental heterogeneity hypothesis emphasize the direct relationship between environmental factors and species richness, yet the influence of environmental factors on species richness is not always direct. Especially at small scales, the environmental influences on species richness are achieved indirectly through biological factors [37]. Therefore, to ascertain the relationship between species richness and environmental factors, it is necessary to study the relationship between species richness and biological factors. Taylor et al. [38] proposed a theory to explain the variation in species richness, i.e., the species pool hypothesis. According to the hypothesis, the size of the species pool and the evenness of the species in the species pool at a local scale has a direct impact on the species richness of local communities [37]. The number of species in a plant community will increase as the number of individuals or areas increases. Initially, the rate of increase in the number of species is very fast and then gradually slows down [37, 39]. Therefore, the evenness of biological factors and the total number of individuals in the community also affect the spatial variation of species richness.

On a large scale, many studies report that biomass is determined by precipitation and temperature. However, it has been shown that the biomass of desert areas is comparatively less affected by climatic factors than temperate grasslands and shrublands [40, 41]. On the medium-small scale, in addition to climatic factors, environmental factors such as small topography and microclimate conditions directly impact the community structure and species types, thereby indirectly affecting vegetation biomass. Moreover, environmental factors such as precipitation and temperature influence species richness and biodiversity to varying degrees [38–46]. Therefore, it is assumed that climate factors (especially precipitation) are the main factors driving biomass and species richness in desert areas. In order to verify this hypothesis, 4,500 sample quadrats were set up in the desert area of northern Xinjiang. Using vegetation ecology and quantitative ecology methods, redundancy analysis (RDA) and structural equation modeling (SEM) were carried out to investigate the spatial distribution characteristics of biomass and species richness in the desert area and to identify the factors driving biomass and species richness variations. The objectives of the present study were threefold: (1) to assess the status of biomass and species richness in the desert area and their spatial distribution characteristics; (2) to elucidate the main factors driving the spatial variations in biomass and species richness; and (3) to analyze the coupling relationship between biomass and species richness. The study aims to provide a scientific basis for the evaluation of ecological restoration measures, desert ecosystem management, and sustainable development.

## Materials and methods

### Study area

**Fig 1:**
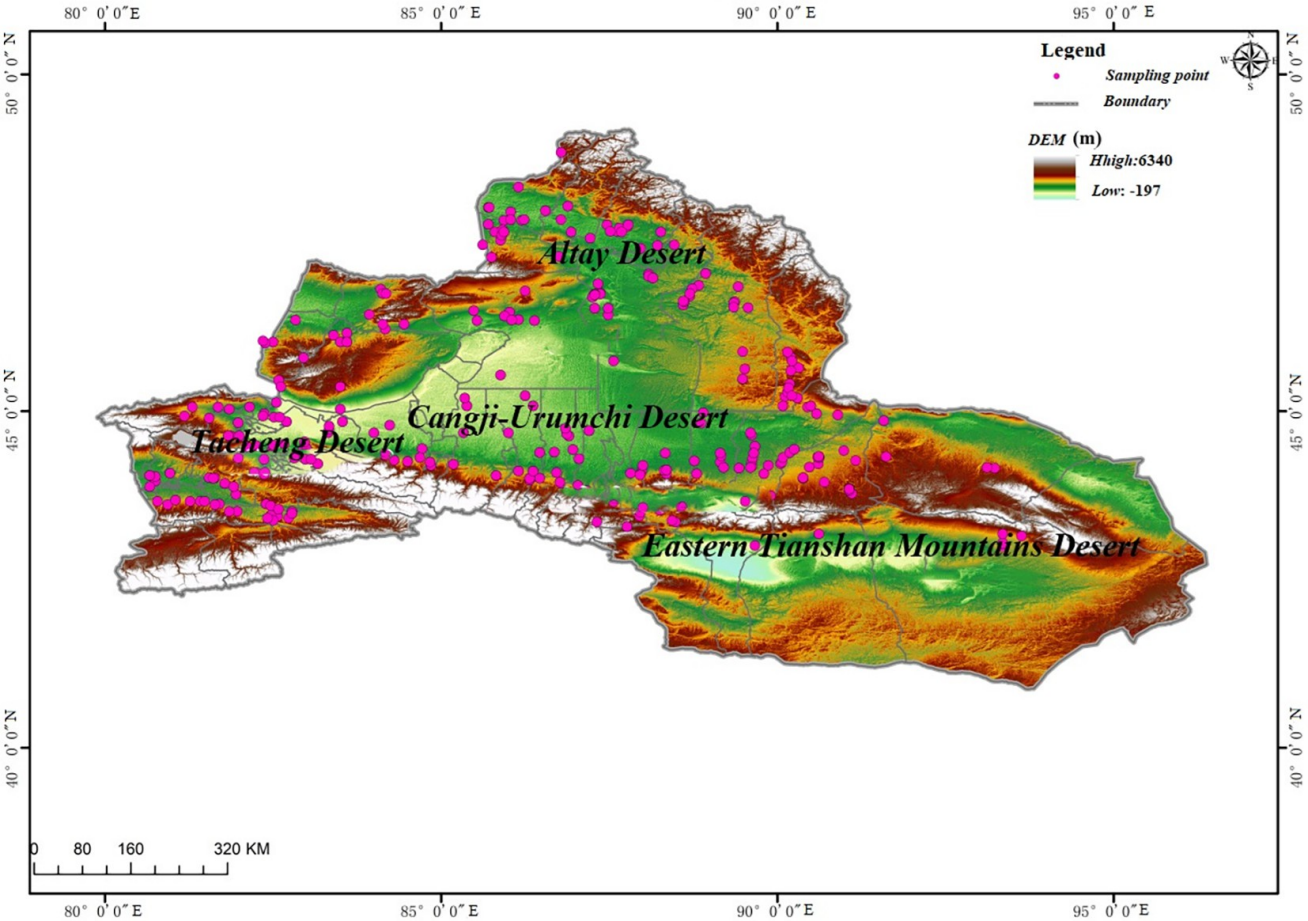
Spatial distribution of the plots in the study area.

The study area (41.1°–49.3°N and 79.8°–91.6°E) is located in the desert area of northern Xinjiang, which has a total area of 4500 × 10^4^ hm^2^ (Fig 1). We used a combination of specific route survey and fixed-point sampling to collect samples. The study area includes the deserts in Junggar Basin, Altay, Tacheng, Yili, Bole, Nuomin Gobi, and Tuha Basin. The average annual temperature in the deserts of Junggar Basin, Altay and Tacheng, and East Tianshan are 5–7.5°C, 2.5–5°C and 9.8–13.9°C, respectively. The overall precipitation in northern Xinjiang shows a pattern of more precipitation in the west than in the east, and more precipitation in the basin edge than in the basin center. The precipitation in the western edge of the Junggar Basin is 200–250 mm, the north-south edge is about 200 mm, and the center of the basin is about 100–150 mm. The precipitation in the deserts of the eastern Xinjiang is about 34.6 mm, and the vertical changes in precipitation are significant. Moreover, the soil shows a horizontal belt pattern from north to south. The soil in Tacheng Basin and most of the northern part of the Junggar Basin is brown soil, which changes to light brown soil in the south. In the Yili Valley, the soil is mainly sierozem soil.

The distribution of vegetation in the desert areas is as follows: *Haloxylon ammodendron*, *Reaumuria soongorica*, and *Anabasis aphylla* are largely distributed in the southwestern part of the Junggar Basin. *Artemisia gracilescens* is widely distributed in Tacheng and Bole Valley. *Artemisia kaschgarica* and *Stipa capillata* are mainly distributed in the Yili and Tacheng areas. *Anabasis brevifolia* and *Nitraria sphaerocarpa* are distributed in the desert areas of the East Tianshan. *Nanophyton erinaceum* and *Anabasis salsa* are distributed in large areas in the northern Junggar Basin. The difference in precipitation between the regions and the significant vertical changes in precipitation in mountainous areas have led to obvious differences in the desert regions and zonal distribution characteristics. According to the landforms, soil types, climatic characteristics, and vegetation distribution in northern Xinjiang, the study area can be divided into four desert regions: Altay Desert, Tacheng Desert, Changji-Urumqi Desert, and East Tianshan Desert [2].

### Experimental design

**Fig 2:**
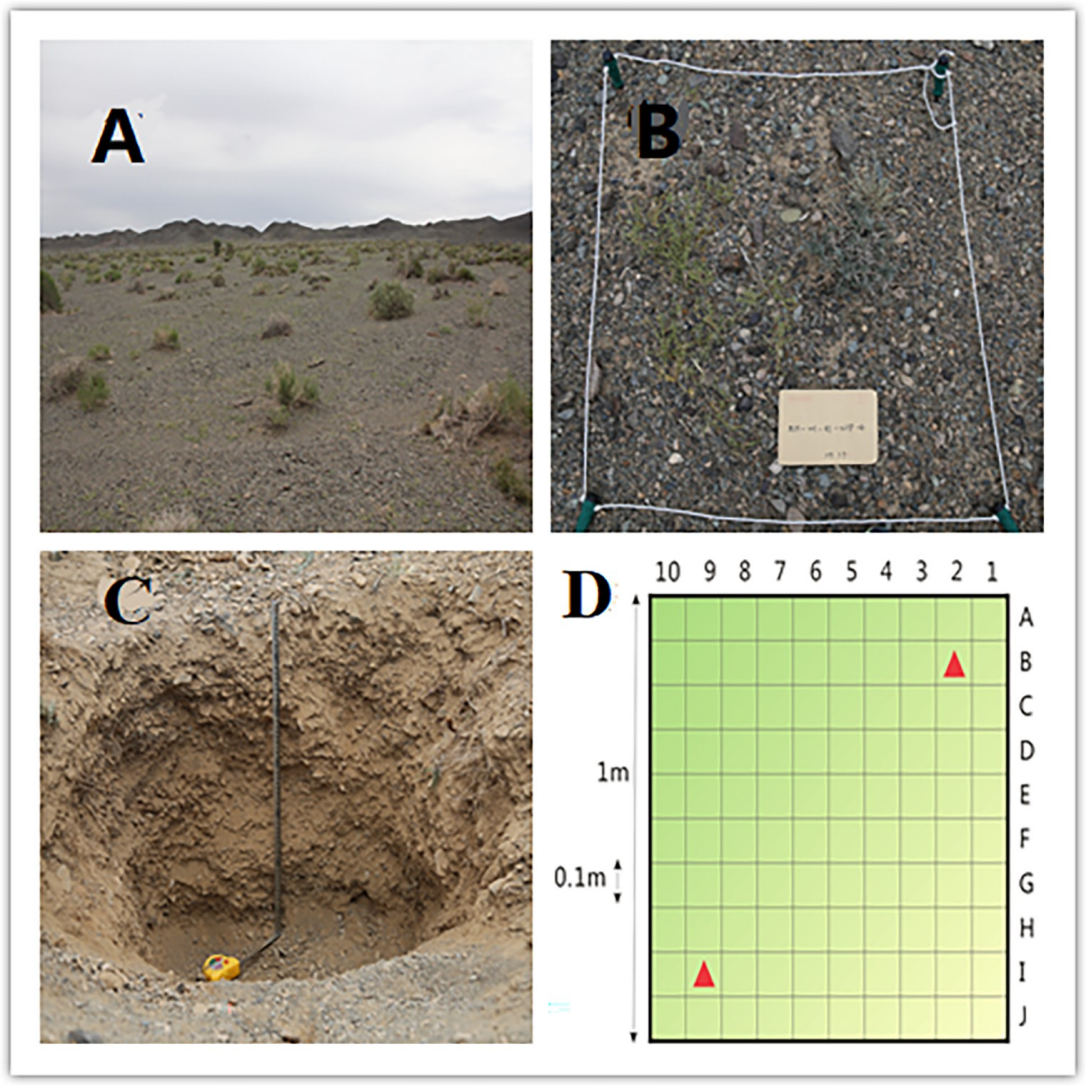
Quadrat design and soil profile.

A field survey of desert communities in northern Xinjiang was conducted using conventional quadrat sampling methods, and the primary community types were determined using the dominant species and importance value [30]. Between the summer months of July and September in 2017–2019, 527 sampling points (9 quadrats at each sampling point, i.e., a total of ~4,500 quadrats) were selected (Fig 1). Specifically, 114 sampling points were in the Altay Desert, 239 in the Tacheng-Yili Desert, 132 in the Changji-Urumqi Desert, and 42 in the East Tianshan Desert. The quadrats were selected in areas with relatively low human interference and flat terrain. If the topography of a certain area was complex, more quadrats were used to ensure the comprehensiveness of the sampling. Moreover, the desert plant samples in the selected quadrats were representative of the area. In each area, 4 strips of 100 m in length were selected, the distance between the strips was more than 50 m, and one 1 m × 1 m quadrat was selected at 25-m intervals (Fig 2B and 2D). Each quadrat was divided into 100 grid units (A1-J10, 0.1 m × 0.1 m; Fig 2D).

### Field survey and sampling

The field survey was first conducted in the desert area of East Tianshan, followed by the Junggar Basin, Altay, and Tacheng-Yili. In order to estimate the biomass and species richness and associated distribution characteristics of the desert plant communities in northern Xinjiang, the latitude and longitude, elevation, and community name of each sampling point were recorded during the field survey. At the sampling site, five 10 m × 10 m shrub quadrats were set according to the plum blossom sampling method, and the new branches of all of the species were cut off and bagged. Moreover, three cross-sections with equal distances were established, and each cross-section had three herbal quadrats (1 m × 1 m). For the aboveground parts of the plants, the quantitative characteristics of the vegetation were recorded, as well as the species in the quadrat. The vegetation coverage of the shrub quadrats was measured visually, and the vegetation coverage of the herb quadrats was measured using the needle-puncture method. The community coverage (%) of each quadrat was estimated, and the height and density (plants/m^2^) of the plants were measured. Then, the number of appearances of each species in the quadrat was used to calculate the frequency. Then the aboveground parts of the plants in each quadrat were sampled, and the fresh weights (g/m^2^) of the plants were weighed. After collecting all of the species in the herb quadrat, the surface litter and standing litter were collected and weighed (g/m^2^) before bagging. They were brought back to the laboratory, wrapped in paper, and placed in an oven at 105°C for 30 min for high-temperature curing, and then at 80°C for 24 h for drying. After that, the dry weight (g/m^2^) was measured. After measuring the AGB of the herb and shrub quadrats, the underground parts were observed and BGB was measured. Specifically, a 1 m × 1 m soil profile was established in the center of the quadrat, and the soil was sampled at different depths with a soil sampler (0–5 cm, 5–10 cm, 10–20 cm, 20–30 cm, 30–50 cm, 50–70 cm, and 70–100 cm) (Fig 2C). Then, the soil root samples from the same depth of each quadrat were mixed and placed in a nylon bag (mesh size: 2 mm). The root samples were then cleaned in the laboratory.

### Data analysis

#### Calculation of species richness

First, the importance value (IV) of the species was calculated based on the height, coverage, frequency, and density of shrubs and herbs in each quadrat:

$$\text{Importance}\;\text{value}\;\left(\text{IV}\right)=\left(\text{relative}\;\text{height}+\text{relative}\;\text{coverage}+\text{relative}\;\text{density}+\text{relative}\;\text{biomass}\right)/4.$$ (1)

Then, the species richness index was calculated according to the importance value of species in the quadrat:

$$\text{Patrick}\;\text{index}\;\left(R_{p}\right):\;R_{p}=S,$$ (2)

where *S* is the average number of species in the quadrat.

#### Environmental data

GPS data were used to obtain the latitude and longitude coordinates and elevation of each quadrat. The high-resolution (1 km × 1 km) climate data, which are widely used in studies of the distribution of species diversity, were obtained from the world climate database WorldClim (www.worldclim.org/). The "Extract Multi Values To Points" module in *arcpy* was used to extract the average temperature and precipitation raster data in the multi-temporal world climate database. Then, the ArcGIS spatial link function was used to obtain the precipitation and temperature of the quadrats in different desert areas.

#### Data analysis

The inverse distance weighted spatial interpolation method is based on the similarity of the sample points in the interpolation area. The weighted average value of the sample points in the adjacent area was calculated to estimate the value of the cell, and then a surface was obtained by interpolation and is a weighted average interpolation method. When the data in the whole study area are relatively evenly distributed and there is no clustering, the effect of the inverse distance weighted spatial interpolation method is optimal. It is believed that the closer the distance between two objects, the higher the similarity, and vice versa [48]. The inverse distance weighted algorithm of the Geostatistical Wizard module of ArcGIS 10.4.1 was used to perform the interpolation.

A Kolmogorov-Smirnov test was used to test the normality of biomass and richness distribution, and Pearson’s correlation coefficient (r) was used to determine the relationship between biomass and species richness of the desert plant communities and climate variables (SPSS 21.0). Moreover, single-factor analysis of variance and multiple comparisons were performed to test the differences in biomass and species richness among different desert areas. The original biomass and richness data in the study area were approximated as a right-skewed distribution, and thus *log10* transformation was performed on the original data before interpolation. Inverse distance weighted Kriging interpolation was carried out using the geostatistical analysis tool in ArcGIS 10.4.1. The spatial distributions of biomass and species richness of the desert communities in northern Xinjiang were obtained. RDA was performed using R3.6.2 software. RDA is constrained principal component analysis. Its purpose is to find new variables to replace the original variables, and its advantage is that it considers the influence of environmental factors on the quadrat because sometimes we want to obtain the distribution of species under certain conditions (such as altitude). Which species are affected by specific environmental factors? It can be determined that some vegetation is affected by altitude and some by climate factors. Specifically, multiple linear regression analysis was conducted between the response variable matrix and the explanatory variable matrix. The ranking of constraints method was used to directly add explanatory variables to evaluate the influence of the environmental variables and biological variables on biomass and species richness. SEM (structural equation modelling) is a multivariate statistical technique combining factor analysis and path analysis. Its strength lies in the quantitative study of the interaction between multiple variables. Lastly, SEM analysis (Amos 21.0, Chicago, USA) was performed to explore the direct and indirect influences of environmental variables on the biomass and species richness of desert plants.

## Results

### Spatial variation in plant biomass and species richness in northern Xinjiang

Tables 1 and 2 shows the results of AGB, BGB, litter biomass, community richness (*Patrick*) and species richness by Functional groups (Shrub, Sub shrub, Perennial herb and Annual herb) in the four desert areas in northern Xinjiang.

**Table 1:** Descriptive statistics of biomass, litter, and species richness of desert grasslands in northern Xinjiang.

Desert	Variable	Mean	Med.	SD	Ske.	Kur.	Min.	Max.	CV (%)
Altay Desert	*AGB*	133.62	124	99.42	1.819	5.635	2	708.12	74.41
	*BGB*	979.25	946	256.23	1.778	6.126	405	2105.4	26.15
	*litter*	18.04	16	8.82	1.897	4.734	5	55.35	48.89
	*Patrick*	83	79	52.49	1.662	6.853	0	377	63.25
Tacheng Desert	*AGB*	143.54	102	114.12	2.338	4.88	13	632.21	79.50
	*BGB*	952.65	613.5	592.39	1.855	3.2	458.6	3537.3	62.18
	*litter*	13.39	8.83	11.23	1.709	2.421	2	56.46	83.86
	*Patrick*	71	61	49.09	0.895	1.029	0	286	69.01
Changji-Urumqi Desert	*AGB*	127.35	109	48.293	1.827	6.518	2	488.15	37.89
	*BGB*	1045.82	936.84	467.14	1.885	4.94	134.8	2989.2	44.69
	*litter*	12.66	11.195	6.122	1.716	5.924	3	48.75	48.36
	*Patrick*	54	45	43.06	1.917	5.618	1	260	79.74
East Tianshan Desert	*AGB*	57.17	48.08	18.835	2.347	5.761	36	144.12	32.96
	*BGB*	721.37	637.3	228.73	1.009	1.397	120.3	1624.2	31.62
	*litter*	8.168	7.02	5.517	1.67	4.976	2	32	67.54
	*Patrick*	43	29	35.67	2.163	5.96	10	196	83.33

**Table 2:** Descriptive statistics of species richness by functional groups of desert grasslands in northern Xinjiang.

Desert	Functional groups	Mean	Med.	SD	Ske.	Kur.	Min.	Max.	CV (%)
Altay Desert	Shrub	7.6	6	6.34	1.99	4.13	3	20	0.83
	Sub shrub	4.4	3	2.33	2.09	4.42	3	9	0.53
	Perennial herb	8	7	3.61	1.88	4.15	5	18	0.45
	Annual herb	4	2.5	3.24	1.32	1.78	1	12	0.81
Tacheng Desert	Shrub	5.14	4	3.15	2	5.69	1	15	0.61
	Sub shrub	4.21	5	2.52	1.96	3.92	2	12	0.60
	Perennial herb	5.57	5	3.33	1.52	4.22	0	15	0.60
	Annual herb	11.5	10	3.73	1.36	2.42	7	22	0.32
Changji-Urumqi Desert	Shrub	4.11	4	2.08	1.53	2.53	2	9	0.51
	Sub shrub	2.58	2	1.85	2.27	6.04	1	8	0.71
	Perennial herb	5.15	4	3.63	1.53	4.39	0	15	0.71
	Annual herb	9.5	8	4.39	1.32	1.37	5	18	0.46
East Tianshan Desert	Shrub	3.5	2.5	2.46	1.6	1.12	0	9	0.70
	Sub shrub	3.5	3	2.45	1.43	2.42	1	9	0.70
	Perennial herb	2.5	2	1.8	1.05	2.47	0	6	0.72
	Annual herb	3	3	2.62	1.6	4.07	1	9	0.65

Table 1 shows the results of AGB, BGB, litter biomass, and species richness (*Patrick*) in the four desert areas in northern Xinjiang. The average values of AGB, BGB, *litter*, and *Patrick* in the Altay Desert were 133.62 gm^−2^, 979.25 gm^−2^, 18.04 gm^−2^ and 83, and the coefficients of variation (*CV*) were 74.41%, 26.15%, 48.89% and 63.25%, respectively. In the Tacheng Desert, the average AGB, BGB, *litter*, and *Patrick* were 143.54 gm^−2^, 952.65 gm^−2^, 13.39 gm^−2^ and 71, with *CV* values of 79.50%, 62.18%, 83.86% and 69.01%, respectively. In the Changji-Urumqi Desert, the average values of AGB, BGB, *litter*, and *Patrick* were 127.35 gm^−2^, 1045.82 gm^−2^, 12.66 gm^−2^, and 54, with *CV*s of 37.89%, 44.69%, 48.36%, and 79.74%, respectively. Lastly, in the East Tianshan Desert, the average AGB, BGB, *litter*, and *Patrick* were 57.17 gm^−2^, 721.37 gm^−2^, 8.168 gm^−2^ and 43, and the *CVs* were 32.96%, 31.62%, 67.54% and 83.33%, respectively. The average and median values of AGB, BGB, *litter*, and *Patrick* in the Altay, Tacheng and Changji-Urumqi Deserts were significantly higher than those of the East Tianshan Desert, showing a significant decreasing trend from west to east.

Table 2 shows the results of species richness by functional groups (shrub, sub shrubs, perennial herbs and annual herbs) in the four desert areas in northern Xinjiang. The average values of species richness (Patrick) in the Altay Desert by functional groups were 7.6, 4.4, 8, and 4, respectively, and the coefficients of variation (CVs) were 83%, 53%, 45%, and 81%, respectively. In the Tacheng Desert, the average species richness (Patrick) by functional group was 5.14, 4.21, 5.57, and 11.5, with CV values of 61%, 60%, 60% and 32%, respectively. In the Changji-Urumqi Desert, the average values of species richness (Patrick) by functional group were 4.11, 2.58, 5.15 and 9.5, with CVs of 51%, 71%, 71% and 46%, respectively. Last, in the East Tianshan Desert, the average species richness (Patrick) by functional groups was 3.5, 3.5, 2.5 and 3, and the CVs were 70%, 70%, 72% and 65%, respectively. The average and median values of species richness (Patrick) in the Altay, Tacheng and Changji-Urumqi deserts were significantly higher than those in the East Tianshan Desert, showing a significant decreasing trend from west to east.

Moreover, because the *CV* values were all between 10%–100% [45], this indicated that AGB, BGB, *litter*, and *Patrick* had moderate spatial variation. With the exception of the Tacheng Desert, where the *CV* of BGB was lower than that of AGB and *litter*, the *CV* values of BGB of all of the deserts were higher than those of AGB and *litter*, indicating that the spatial variability of BGB was higher than that of AGB and *litter* biomass. The *CV* values of *Patrick* in the Changji-Urumqi and East Tianshan deserts were higher than those of the Altay and Tacheng Deserts. The *CV* values of *Patrick* showed a decreasing trend from east to west. The spatial variation in species richness in the East Tianshan and Changji-Urumqi Deserts was significantly higher than that of the Altay and Tacheng deserts (*P*<0.01), and the skewness coefficients (*Ske*) of the East Tianshan and Changji-Urumqi Deserts were also relatively higher than those of the Altay and Tacheng Deserts, indicating that the species richness of the Altay and Tacheng Deserts was relatively stable, and the degree of spatial variation was small.

The value of Table 3 of the data is the average value. Table 3 shows that the aboveground biomass, underground biomass and total biomass (TB) of grassland in the Altay and Tacheng-Yili desert areas were higher than those in the Changji-Urumqi desert area and Eastern Xinjiang desert area. The TB in Zhaosu, Xinyuan, Gongliu, Jimunai, Altay, Changji and Urumqi is relatively high, while the TB in Shanshan and Mulei is relatively low. The AGB was relatively high in Tuoli, Jinghe, Zhaosu, Gongliu, Jimunai, Altay and Urumqi but relatively low in other regions. BGB was relatively high in Zhaosu, Xinyuan, Jimunai, Altay and Urumqi and relatively low in other regions.

**Table 3:** Spatial variability of aboveground biomass, underground biomass and total biomass of desert in northern Xinjiang.

Desert	Sample	AGB	BGB	Total biomass
Altay Desert				
Aletai	45	205.5±7.8a	1415.12±234.3a	1620.6±245.5a
Fuhai	32	76.55±5.5b	712.38±72.8b	788.55±60.2b
Jimunai	40	212.8±20.2a	1213.68±252.5a	1532.8±305.5a
Fuxun	42	86.54±4.9b	625.05±73.8b	711.54±41.8b
Tacheng Desert				
Tuoli	20	184.24+22.5a	658.2±54.5a	842.4±64.5a
Jinghe	25	169.1±8.3ba	592.5±45.24a	761.6±52.4a
Tacheng	23	85.72±6.5b	718.8±73.3a	794.5±71.6a
Zhaosu	32	125.3±11.2c	1814.22±105.5b	1939.0±324.5b
Kelamayi	21	193.8±25.3a	617.9±68.24a	811.7±65.5a
Xinyuan	19	78.2±5.9c	2039.9±236.25b	2118.0±311.2b
Gongliu	24	229.9±20.3a	788.85.±74.3a	1018.7±114.5c
Changji-Urumqi Deser				
Urumqi	34	174.4±20.3a	1315.21±145.2a	1319.6±342.5a
Cangji	32	90.67±12.5b	1188.55±134.1a	1115.6±105.2a
Hutubi	24	87.7±13.5b	705.1±45.8b	792.8±44.3b
East Tianshan Desert				
Hami	26	74.24±16.1a	849.25±102.5a	923.49±84.7a
Qitai	20	81.8±6.6a	813.12±321.8a	894.9±142.5a
Balikun	30	147.6±12.0b	773.72±62.45a	921.32±72.3a
Mulei	18	182.57±21.2c	467.55±52.52b	656.79±80.1b
Shanshan	20	82.69±4.6a	566.55±42.52b	649.24±52.1b

Table 4 presents the species richness data according to the classification of functional groups; the sum of the mean values of major desert community types was taken. Table 4 shows that overall, the species richness indices of perennial and annual herbs were relatively high in the Altay Desert, Tacheng Desert and Changji-Urumqi Desert and were higher than those of shrubs and semishrubs. The species richness index of desert shrubs and semishrubs in the East Tianshan Mountains was higher than that of perennial and annual herbs. The highest species richness index was 176 for annual herbs in the Tacheng Desert area. The lowest value was found for the perennial herbaceous species in the eastern Tianshan Desert area, with a richness index of 15. The Tacheng Desert is hot in summer and cold in winter and short in spring and autumn. The average annual precipitation is 100–200 mm, and the desert is mainly a semishrub desert. Due to the high precipitation in winter and spring, desert vegetation generally develops short-growing plant lamella and perennial short-growing plant lamella, and annual herbs also develop in a large area. The eastern Tianshan Desert hydrological network is very underdeveloped, with only intermittent rivers and annual precipitation of only 20–70 mm; thus, it is very arid and has mainly shrubs and subshrubs. Plant composition is extremely poor, and most of the ancient species are endemic to central Asia.

**Table 4:** Patrick index of different plant communities life-forms in northern Xinjiang.

Desert	Desert community types	Functional groups	Species richness(Patrick)
Altay Desert	Form.*Artemisia frigida* Form.*Anabasis salsa* Form.*Haloxylon ammodendron* Form.*Ceratoides latens* Form.*Artemisia arenaria*	Shrub	38
		Sub shrub	22
		Perennial herb	96
		Annual herb	48
Tacheng Desert	Form.*Kochia prostrata* Form.*Reaumuria soongorica* Form.*Seriphidium borotalense* Form.*Haloxylon ammodendron* Form.*Nanophyton erinaceum* Form.*Ceratoides latens*	Shrub	78
		Sub shrub	40
		Perennial herb	80
		Annual herb	176
Changji-Urumqi Desert	Form.*Haloxylon ammodendron* Form.*Ceratoides latens* Form.*Nanophyton erinaceum* Form.*Seriphidium transillense* Form.*Reaumuria soongorica* Form.*Petrosimonia sibirica*	Shrub	37
		Sub shrub	31
		Perennial herb	67
		Annual herb	57
East Tianshan Desert	Form.*Ephedra przewalskii* Form.*Iljinia regelii*	Shrub	35
		Sub shrub	28
		Perennial herb	15
		Annual herb	17

### Distribution of plant biomass and species richness in different deserts

**Fig 3:**
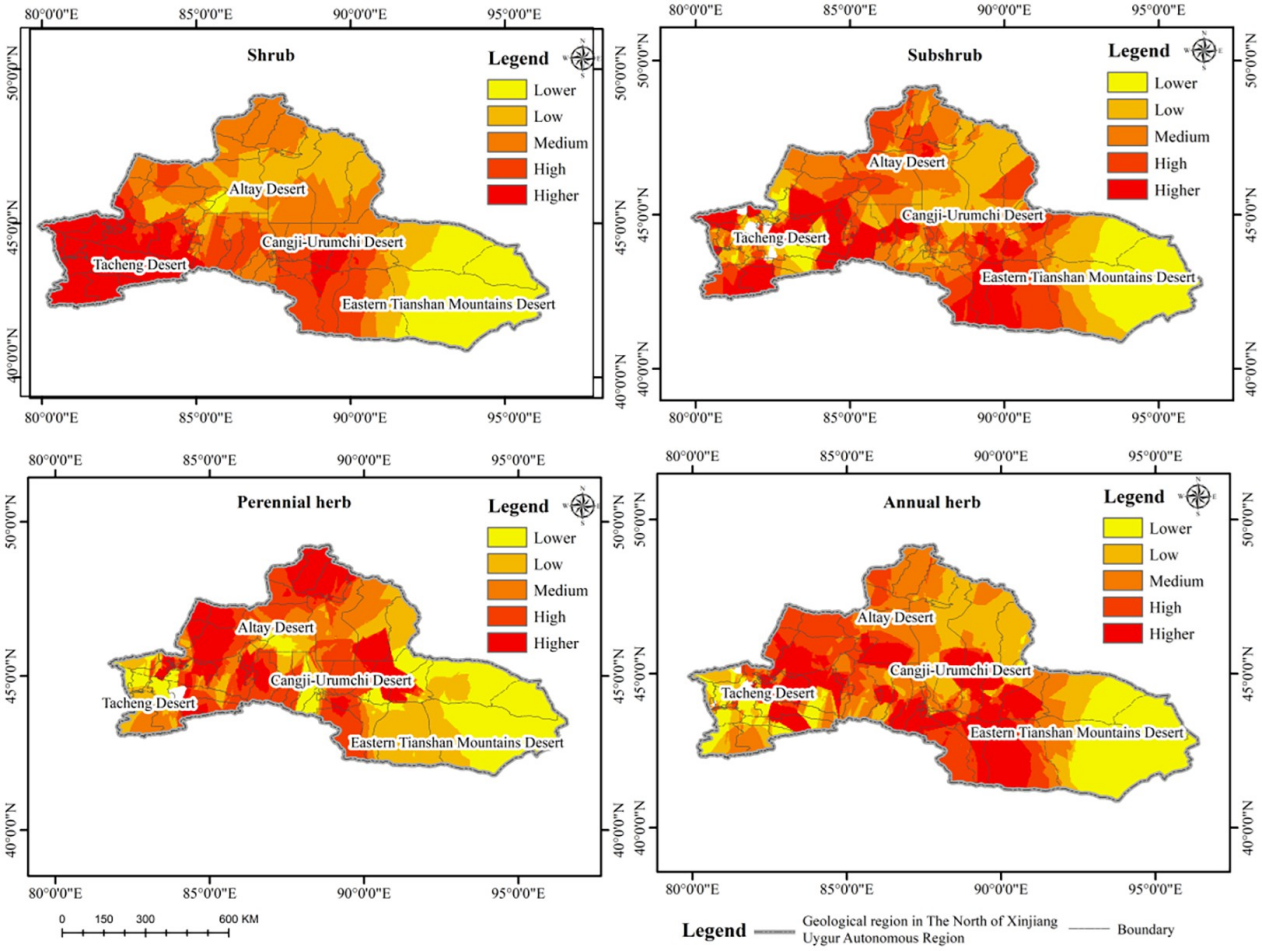
Spatial pattern of biomass in northern Xinjiang.

The spatial distribution of plant biomass in different desert areas of northern Xinjiang was similar, and the spatial variation in the research variables in each desert area is shown in Fig 3. Generally, due to the complexity and spatial heterogeneity of climate, elevation, and soil material and the influence of human activities, the plant biomass of the Tacheng, Yili, Altay, and Changji-Urumqi Deserts was significantly higher than that of the East Tianshan Desert area (*P*<0.01). The biomass gradually decreased from west to east, with certain differences observed in some areas. High values of biomass were mainly distributed in the desert areas of Tacheng, Yili, Changji, and Urumqi and the west of Mulei Dashigu where there is abundant rainfall, and the low values were distributed in most areas of the East Tianshan Desert.

**Fig 4:**
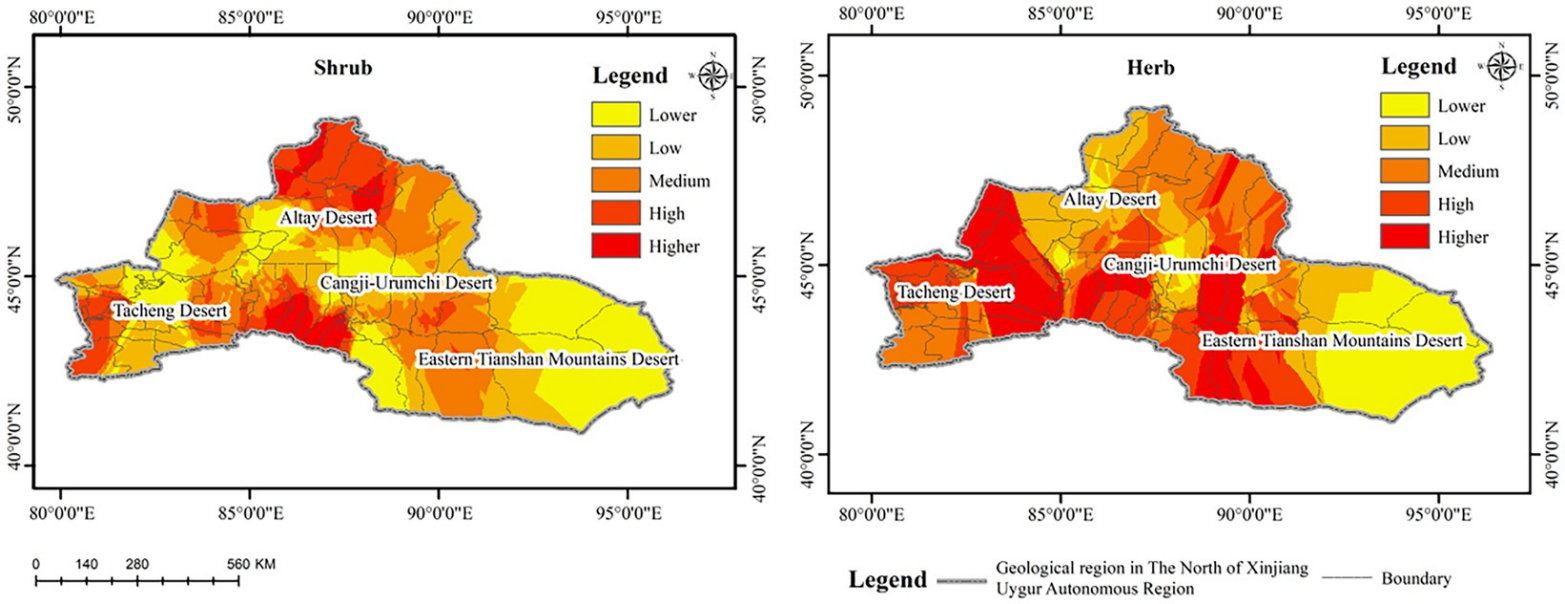
Spatial pattern of species richness in northern Xinjiang.

The spatial distribution of shrubs and herbs in the northern Xinjiang desert areas differed significantly. The spatial variation in the research variables in each desert area is shown in Fig 4. Due to the influence of climate, elevation, and biological factors, particularly climatic factors, the highest richness of shrubs was observed in Tacheng, Yili, Bortala Mongolian Autonomous Prefecture, Changji, Urumqi, and the west of Mulei Dashitou. In most areas of the Altay Desert, the shrub richness was mostly at a moderate level, with higher values northwest of Fuyun County and the southern part of Qinghe County. The highest value of herb richness was observed in Zhaosu and Tekesi in the south of Yili Kazakh Autonomous Prefecture, Huocheng County in the middle, and Haba River, Burqin, and Jeminay counties in the Altay Desert, and Changji, and Urumqi.

Overall, the biomass and species richness of desert plants in northern Xinjiang showed a gradual decreasing trend from west to east. Under the influence of the westerly circulation and the humid airflow from the west in the western part of Junggar and Tacheng-Yili and the northern slope of the Tianshan Mountains in the west of Mulei Dashitou, the amount of precipitation increases with the increase in elevation, and the maximum precipitation can reach 600–800 mm. Moreover, the biomass and species richness were significantly higher than that in the East Tianshan Desert. The reason for the low biomass and species richness in the East Tianshan Desert was because the area was under the influence of the Mongolian high-pressure anticyclone divergence field, which resulted in low precipitation and strong evaporation. The annual precipitation is below 30 mm, the dryness is above 16 (as high as 60 in some areas), and the desert vegetation is sparse, with an average coverage of less than 5%.

### Factors driving plant biomass and species richness in the desert areas

#### RDA analysis

**Fig 5:**
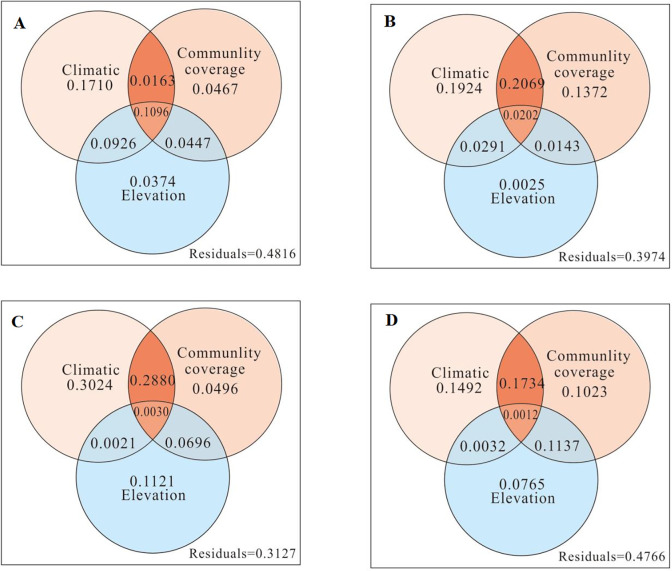
Redundancy analysis of factors driving the spatial variation in desert biomass in northern Xinjiang.

**Fig 6:**
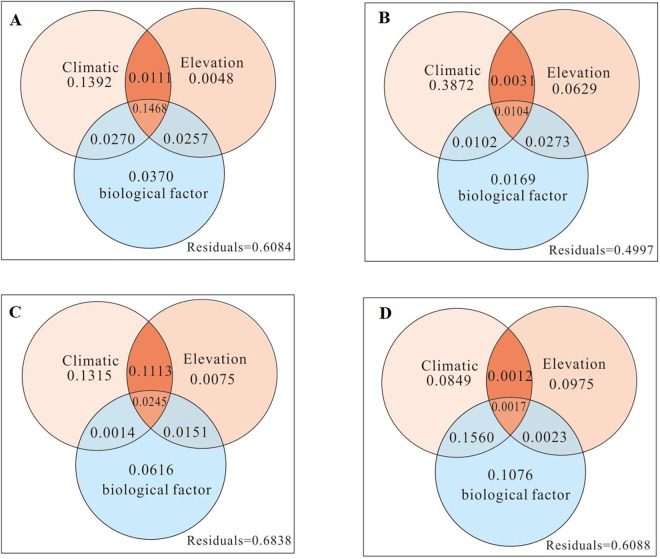
Redundancy analysis of the factors driving the spatial variation of desert species richness in northern Xinjiang.

In order to further investigate the influences of climate factors, plant community characteristics (community coverage), biological factors (species evenness and total number of communities), and elevation on the spatial variations in biomass and species richness in northern Xinjiang, RDA was carried out. The results showed that the explanatory power of various environmental factors on the spatial variation of biomass and species richness of the Altay Desert (Figs 5A and 6A) was 51.84% and 39.16%, respectively. The explanatory power for the Tacheng-Yili Desert (Figs 5B and 6B) was 60.26% and 50.03%, respectively. For the Changji-Urumqi Desert (Figs 5C and 6D), the explanatory power was 68.73% and 39.12%, respectively. For the East Tianshan Desert (Figs 5D and 6C), the explanatory power was 52.34% and 31.62%, respectively. Among the environmental factors, climate factors were the main factors driving the biomass and species richness of the desert plant communities in northern Xinjiang. The order of the factors driving biomass was climate factors > community coverage > elevation. One exception was that in the Changji-Urumqi Desert (Fig 5C), the explanatory power of elevation (11.21%) was higher than that of community coverage. The order of factors driving species richness was climate factor > biological factor > elevation. However, in the Tacheng-Yili Desert (Fig 6B), the explanatory power of elevation (6.29%) was slightly higher than that of biological factor.

The Altay desert has high annual precipitation, low temperature, no obvious drought, humid climate and relatively high species richness. The Tacheng-Yili desert has more precipitation in spring and winter, which makes the soil water content higher, but the temperature is relatively low, and the rainfall is lower in autumn. There is more rainfall in summer, but the temperature is high, and the availability of precipitation is reduced. Drought and semidrought mainly occur in this period, and plant growth and development are stagnant. In this climate, the accumulation of plant organic matter in spring was higher than that in autumn, and the cambium layer was formed by the large amount of ephemeral plants and perennial ephemeral plants, forming the desert vegetation type of ephemeral plants. In the Changji-Urumqi desert, there is more rain in spring and summer, less rain in autumn and less snow in winter. Precipitation in winter and spring is beneficial to the development of ephemeral plants and perennial ephemeral plants. Although there is a large amount of precipitation in summer, the temperature is high. Drought and semidrought often occur in this season; thus, it is only suitable for annual plants and perennial plants to grow. In this climate, a large area of small sub shrub desert will be formed, and the accumulation of plant organic matter in spring is much lower than that in summer and autumn. The desert of the East Tianshan Mountains is characterized by high temperature and low annual precipitation, which mainly occurs in summer. In spring and autumn, there is little rain, and the areas are not suitable for the growth and development of ephemeral plants. The accumulation of plant biomass was also concentrated in summer. In this climate environment, desert vegetation mainly consisted of shrubs, shrubs and annual desert plants at the vegetative stage.

Overall, the main factor driving the biomass and species richness of desert plant communities in northern Xinjiang was climate factors. This is consistent with our hypothesis that "climatic factors" are the main drivers of desert community biomass and species richness. The climate factors worked together with community coverage, biological factors, and elevation to influence the biomass and richness. The influence of elevation alone on biomass and richness was relatively low.

#### SEM analysis

**Fig 7:**
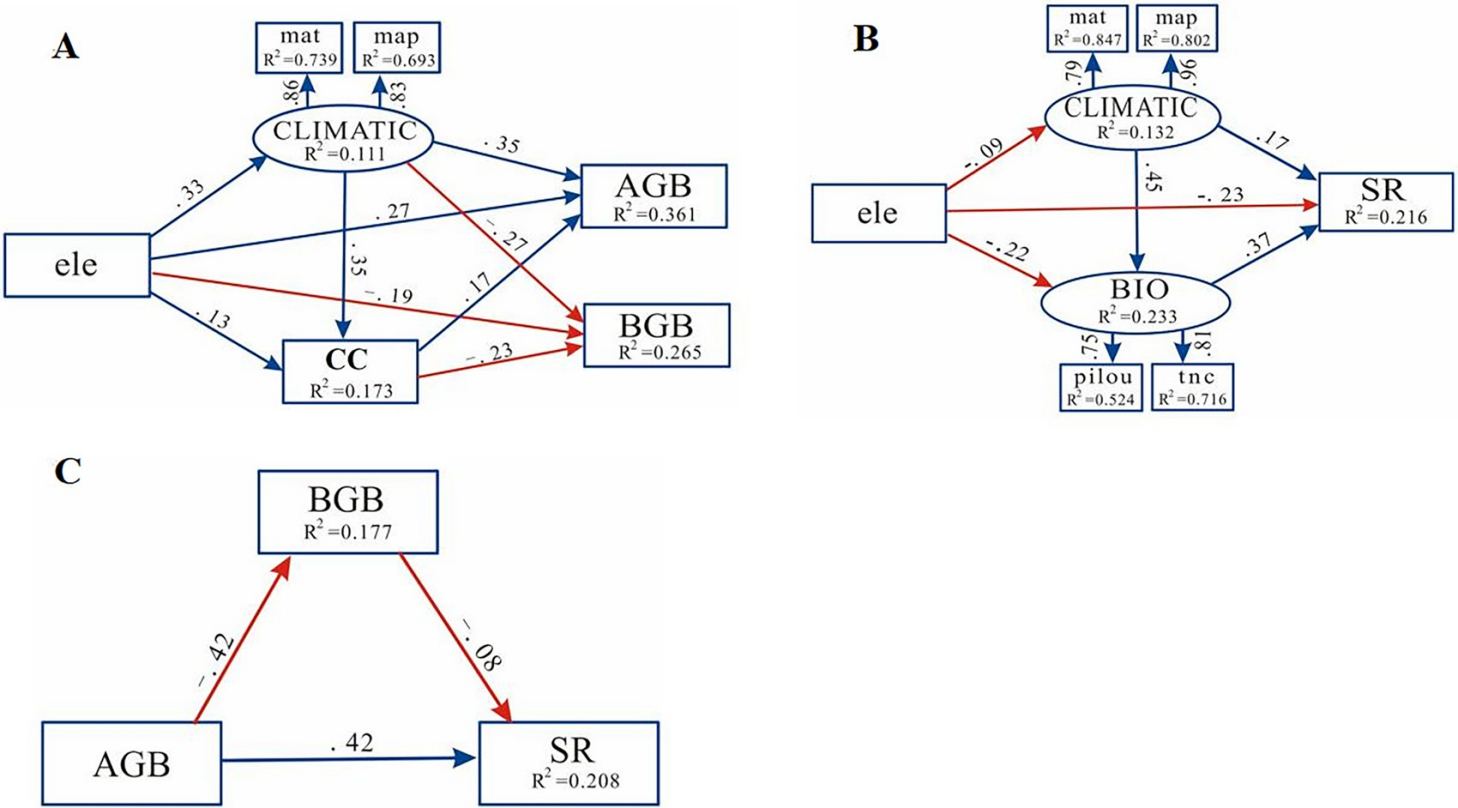
SEM analysis of the factors driving desert plant biomass and richness in northern Xinjiang.

*SEM analysis of the driving factors of biomass*. SEM analysis was carried out to study the effects of climate factors, elevation, and community coverage on the spatial variation of biomass in northern Xinjiang. Based on Fig 7A, it can be observed that climate factors, elevation, and community coverage had a direct positive effect on AGB in northern Xinjiang and a direct negative effect on BGB.

In addition to the direct effects of the above factors, the biomass of the desert plant communities in northern Xinjiang was also indirectly affected by these factors. As the path did not contain the value of 0, climate factors and community coverage (CC) had a mediating effect (Fig 7A and Table 5). For example, upon changing climate and community coverage, elevation(ele) had a significant effect on AGB and BGB (*P*<0.01).

**Table 5:** Factor mediating effect test.

Parameter	Estimate	Lower	Upper	P
ele->climatic->AGB	0.117	0.076	0.171	0.000
ele->climatic->BGB	−0.090	−0.133	−0.056	0.000
ele->CC->AGB	0.047	0.014	0.086	0.004
ele->CC->BGB	−0.036	−0.072	−0.011	0.003
ele->climatic->CC->AGB	0.020	0.010	0.033	0.000
ele->climatic->CC->BGB	−0.026	−0.044	−0.015	0.000

*SEM analysis of the factors driving species richness*. SEM analysis was carried out to evaluate the effects of climate factors, elevation, and biological factors on the spatial variation in species richness in northern Xinjiang. Based on Fig 7B, it is evident that climate factors and biological factors had a direct positive effect on the species richness of northern Xinjiang, while elevation had a direct negative effect.

In addition to the direct effects of the above factors, the species richness of the desert plant communities in northern Xinjiang was also indirectly affected by these factors. As the path did not contain the value of 0, climatic and biological factors had a mediating effect. For example, by individually changing the climatic and biological factors, elevation had a significant effect on species richness (*P*<0.05, Table 6). When elevation changed both the climatic and biological factors, the effect on species richness was non-significant (*P*>0.05, Table 6).

**Table 6:** Factor mediating effect test.

Parameter	Estimate	Lower	Upper	P
ele->CLIMATIC->SR	−0.016	−0.049	0.002	0.050
ele->BIO->SR	−0.082	−0.140	−0.040	0.000
ele->CLIMATIC->BIO->SR	−0.016	−0.041	0.001	0.060

The SEM analysis of driving factors for species richness in the desert in northern Xinjiang also confirmed the "energy and environmental heterogeneity hypotheses". There is a direct correlation between environmental factors and species richness. Environmental factors can affect species richness, but the impact is not always direct. Environmental factors can also indirectly influence species richness by altering biological factors.

### Relationship between the biomass and species richness of desert plants

SEM analysis was carried out to analyze the relationship between biomass and species richness. The results showed that there was a significant positive correlation between AGB and species richness, and there was a non-significant negative correlation between BGB and species richness. The AGB was significantly negatively correlated with BGB (Fig 7C). In addition to the direct impact of AGB, the species richness was also indirectly affected. The path did not contain the value of 0, indicating that AGB affected BGB, which then influenced the species richness. However, the mediating effect was not significant (*P*>0.05, Table 7).

**Table 7:** Factor mediating effect test.

Path	Estimate	Lower	Upper	P
AGB->BGB->SR	0.035	−0.009	0.080	0.131

## Discussion

### Spatial distribution of biomass and species richness in northern Xinjiang

The grasslands in Xinjiang are mainly temperate deserts and temperate steppe deserts, and the total AGB is less than 10% of the national AGB [46]. In the Hexi Corridor desert area in northwestern China, the AGB of grasslands is higher in the northwest and lower in the southeast [47], while the spatial distribution of AGB of typical grasslands in Inner Mongolia is higher in the north and east and lower in the south and west [48]. The herb biomass of temperate desert grasslands in the central part of the Junggar Basin and Beita Mountain in northern Xinjiang increased first with elevation and then decreased, while the biomass of the shrubs gradually decreased with the increase in elevation. The AGB of temperate desert grasslands in the southwestern part of the Junggar Basin is large. The middle section of the northern slope of the Tianshan Mountains, the southwestern part of the Junggar Basin, and the Bole Valley have relatively large BGB, which is significantly higher than other deserts [49]. These earlier results are consistent with the results of this study. Through a field survey of the biomass of the desert plants in northern Xinjiang, a comprehensive understanding of the spatial distribution of the biomass in northern Xinjiang was obtained. According to the spatial distribution of AGB and BGB, the biomass of the Tacheng-Yili Desert, Changji-Urumqi Desert, and Altay Desert was significantly larger than that of the East Tianshan Desert.

Species richness is an important factor influencing biodiversity and is the basis for biodiversity research [50]. Increasing desertification has led to a decrease in species richness in northwest China, which in turn has caused serious ecological problems [51]. According to the spatial distribution of species richness in the Junggar Desert in northern Xinjiang, the Hexi Corridor in Gansu, and the Alxa League in western Inner Mongolia, it was found that the species richness gradually decreased from the Junggar Desert in the west to the Alxa Plateau and the Hexi Corridor in the central and eastern regions, and then increased to the western part of the Inner Mongolia Plateau [51]. The results of the current study are broadly consistent with previous studies. The species richness of the Tacheng-Yili Desert, Changji-Urumqi Desert, and Altay Desert was significantly larger than that of the East Tianshan Desert, showing a gradual decreasing pattern from west to east.

### Factors driving biomass and species richness

The key factor for the formation of grasslands in Xinjiang is climate [20]. According to environmental cybernetics, at a variety of research scales, environmental factors such as climate and physical factors predominantly influence the distribution pattern of plant species diversity [52, 53]. Among various environmental factors, water and energy are the most important factors driving species richness [54]. Studies on the species richness of forests in northeast China have showed that the main factor driving species richness is energy [55]. However, studies on species richness in the northwest desert areas such as Xinjiang Desert and Inner Mongolia grassland have found that water and energy jointly affect species richness [51, 56, 57]. In different regions, the factors driving species richness also differ [58]. The diversity and associated driving factors of three typical plants in the Junggar Desert in northern Xinjiang were studied [59]. It was found that climate factors and latitude played a leading role among all of the environmental factors tested. A study on the factors driving plant species richness in the desert area of northwest China showed that species richness was significantly positively correlated with water and negatively correlated with energy [51]. That study also found that species richness in the entire study area was affected by spatial changes in the water-energy conditions, and species richness gradually decreased from the desert area of northern Xinjiang to the Alxa Plateau and the Hexi Corridor area [51]. The water-energy dynamic hypothesis; that is, that a change in precipitation is the main limiting factor for diversity, has been proposed for the arid area of northwestern China [51]. The results of this study are consistent with those of previous studies. The species richness of northern Xinjiang was mainly affected by climatic conditions, and the species richness gradually decreased from west to east. Climate factors were the main factors driving species richness in northern Xinjiang. By analyzing the relationship between the spatial distribution of species richness and the average annual precipitation and annual temperature in different regions of Xinjiang, Gansu, Inner Mongolia, and Ningxia, it was concluded that the number of desert shrub species increased with the increase in precipitation and decreased with the increase in temperature. In particular, the species richness of Inner Mongolia, Ningxia, and Xinjiang was more greatly influenced by precipitation. With the exception of Inner Mongolia, there was a linear negative correlation between species richness and annual average temperature [60]. A study on the Horqin Desert showed that species richness was strongly affected by precipitation [61]. Moreover, a study on the species richness of deserts in the western, middle, and eastern sections of the northern Tianshan Mountains found that the species richness index in May, July, and September was linearly correlated with annual precipitation, indicating that the species richness of *Artemisia* deserts increased with increased precipitation [62]. The results of this study are broadly consistent with those of previous studies. The richness of shrubs was relatively less affected by precipitation and was relatively stable in desert plants. The species richness of perennial and annual herbs was affected more severely by precipitation. In the study area, there was a positive correlation between the species richness of herbs and precipitation.

Environmental factors are connected and interact with each other. For example, changes in the elevation and slope can result in a change in the temperature and precipitation, thereby indirectly affecting the distribution of AGB of grassland plants [63]. The results of this study showed that climate factors, elevation, and community coverage were extremely significantly positively correlated with AGB. The main driving factor was climate, and elevation, temperature, precipitation, and community coverage had indirect effects on the biomass, which corroborates the results of previous studies. The environmental factors that affect BGB are mainly water (including precipitation and soil moisture), temperature (including air temperature and soil temperature), and light [30]. When the climatic conditions are good, these factors play a positive role in AGB and promote the rapid growth of the aboveground parts of the plant. As a result, BGB decreases. Therefore, precipitation and temperature are negatively correlated with BGB [64, 65]. This study showed that there was a significant negative correlation between precipitation, temperature, elevation, and community coverage and BGB. The reason for this difference may be that in addition to precipitation and temperature, BGB is also influenced by the microenvironment of the sampling sites. On a large scale, temperature and precipitation are important factors affecting biomass, yet on a small scale, biomass is subject to the influences of local climate, topography, and soil factors.

### Relationship between biomass and species richness

A study on the biomass and species richness of 36 *Ephedra distachya* communities, 28 *Seriphidium terrae-albae* communities, and 13 *Artemisia songarica* communities in the Gurbantünggüt Desert showed that the species richness increased significantly with increased plant biomass, reaching the maximum value when the biomass was moderate, thereby showing a unimodal relationship [24]. The results were consistent with that of Chalcraft et al. [66] and Oba et al. [67]. By analyzing the results of about 200 studies on the relationship between biomass and diversity, it was found that, in terms of the type of relationship between species richness and biomass, 30% had a unimodal relationship, 26% showed a positive linear relationship, 12% had a negative linear relationship, and 32% showed a non-significant relationship [68, 69]. In another study, it was found that a linear relationship existed between biomass and the species diversity, richness, and evenness of four different grassland communities in an alpine meadow [70]. A study showed that biomass increased with increased species diversity, and the positive effect intensified year by year [71]. The results of the present study showed that a unimodal relationship existed between the biomass and species richness of desert plants, which is consistent with the results of previous studies.

In general, Desert ecosystem is vulnerable to grassland degradation caused by environmental factors and human activities. Because environment factors include climate factors (e.g., water and heat), and there are many other kinds of environment factors and their interactions are complex, the causal relationship between these factors is not clear. Thus, when we study the correlation between Biomass and species richness and environmental factors in desert grassland in the future, we should include more environmental factors and human factors to explore the influencing factors in a wider dimension and provide scientific basis for the study of sustainable utilization of grassland in desert ecosystem.

## Conclusions

The average AGB, BGB, *litter*, and *Patrick* of the Junggar Desert in northern Xinjiang were 115.42 gm^−2^, 924.77 gm^−2^, 13.06 gm^−2^, and 63 and ranged from 2–708.12 gm^−2^, 120.25–3537.3 gm^−2^, 2–56.46 gm^−2^, and 0–377, respectively, indicating moderate variations. The spatial distribution of biomass and species richness in northern Xinjiang showed a gradual decreasing trend from west to east. The areas with high biomass and species richness were mainly in the Tacheng-Yili Desert, Altay Desert, and Changji-Urumqi Desert.

The results of the RDA analysis on the factors driving desert plant biomass and richness showed that climate factors were primarily driving biomass and species richness in northern Xinjiang. The independent explanatory powers for the spatial variation of biomass and species richness were 20.38% and 18.57% on average.

The SEM results showed that climate factors, elevation, and community coverage had a direct positive effect on the AGB of the desert plant communities in northern Xinjiang, and a direct negative effect on BGB. By altering the climate and community coverage, elevation had a significant effect on AGB and BGB (*P*<0.01). Moreover, climatic and biological factors had a direct positive effect on the species richness of the desert plant communities in northern Xinjiang. However, when elevation changed both the climatic and biological factors, it had a non-significant effect on the species richness in northern Xinjiang (*P*>0.05).

The Eurasian steppe includes the Tibetan Plateau steppe subregion, Mongolian Plateau steppe subregion and Black Sea-Kazakhstan steppe subregion. China’s Inner Mongolia grassland and desert grassland are in the Mongolian Plateau area, and most of the Xinjiang desert grassland areas belong to the Black Sea, China-Kazakhstan grassland area. This study in the northern Xinjiang desert ecological system was the research object, geographically belonging to the Eurasia steppe, desert in Inner Mongolia; in particular, more than 80% of the study areas was in windy rainless arid and semiarid regions. The long winter and livestock grazing and trampling easily lead to wind erosion, desertification and soil erosion, which are similar to the environment and climate of the desert ecosystem in northern Xinjiang. Therefore, this study can play a very important role in the scientific research of AGB, BGB and species richness in the Eurasian steppe and the Inner Mongolia desert in China.

## Supporting information

S1 Fig Spatial distribution of AGB, BGB and TB of plant in northern deserts of Xinjiang. (PDF)

S2 Fig Spatial variation and distribution of AGB of desert in northern of Xinjiang. (PDF)

S1 Table Differences in BGB at different soil depths (0–30 cm) in northern Xinjiang. (PDF)

S2 Table Diversity index of different plant communities life-forms in the Aletai region. **Note:** H-Shannon-Wiener index, D_m_-Mclntosh index, JP-Pielou index, Mc-Mclntosh index, S-Simpson index, Bp-Berger index, R = Patrick index, Me-Menhinick index (the same below).
(PDF)

S3 Table Diversity index of different life-forms in the Yili region. (PDF)

S4 Table Diversity index of different plant communities life-forms in the Bortala region. (PDF)

S5 Table Diversity index of different plant communities life-forms in the east desert subregion of the eastern Tianshan Moutain of Xinjiang. (PDF)

S6 Table Diversity index of different plant communities life-forms in the Tacheng region. (PDF)

S7 Table Diversity index of different plant communities life-forms in the desert subregion of Changji-Urumqi region. (PDF)
