# Supplementary figures and images for: Factors driving the biomass and species richness of desert plants in northern Xinjiang China
Source: PLoS One. 2022 Jul 22;17(7):e0271575. doi: 10.1371/journal.pone.0271575 (PMC9307161; doi:10.1371/journal.pone.0271575)

**S1 Fig.** Spatial distribution of AGB ,BGB and TB of plant in northern deserts of Xinjiang

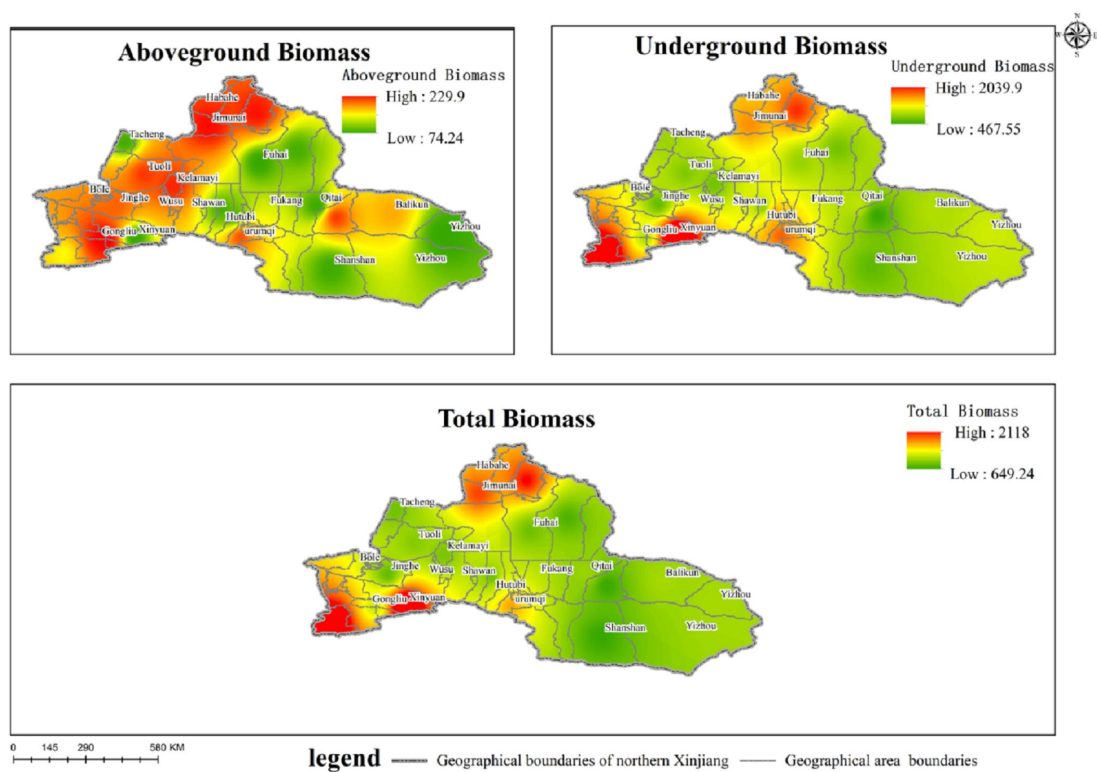

Supplement: S1 Fig — (PDF) [file pone.0271575.s001.pdf]

**S2 Fig** Spatial variation and distribution of AGB of desert in northern of Xinjiang

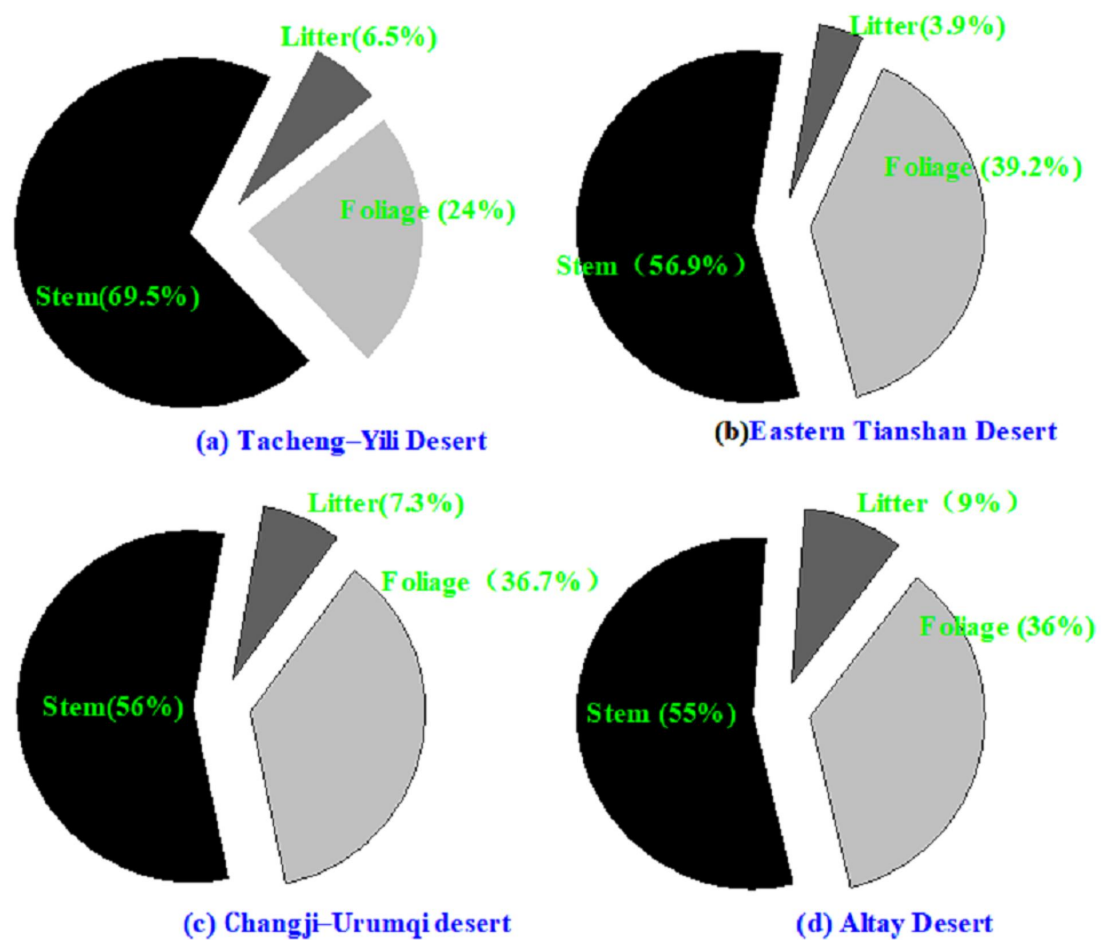

Supplement: S2 Fig — (PDF) [file pone.0271575.s002.pdf]
