# Supplementary material for: Factors driving the biomass and species richness of desert plants in northern Xinjiang China
Source: PLoS One. 2022 Jul 22;17(7):e0271575. doi: 10.1371/journal.pone.0271575 (PMC9307161; doi:10.1371/journal.pone.0271575)
Supplement: S1 Table — (PDF) [file pone.0271575.s003.pdf]

S1 Table Differences in BGB at different soil depths (0–30 cm) in northern Xinjiang

**Soil depth (cm) (Altai Mountain Desert)**	**BGB ($g•m^{-2}$)**	**Soil depth (cm) (Changji–Urumqi Mountain Desert)**	**BGB ($g•m^{-2}$)**
0-5	1174.67	0-5	997.98
5-10	987.95	5-10	721.66
10-20	681.44	10-20	542.84
20-30	570.99	20-30	358.71
**Soil depth (cm) (Tacheng–Yili Mountain Desert)**	**BGB ($g•m^{-2}$)**	**Soil depth (cm) (Eastern Tianshan Mountain Desert)**	**BGB ($g•m^{-2}$)**
0-5	2753.22	0-5	1090.37
5-10	1366.4	5-10	832.62
10-20	1029.88	10-20	634.19
20-30	759.98	20-30	495.96
