# Supplementary material for: Factors driving the biomass and species richness of desert plants in northern Xinjiang China
Source: PLoS One. 2022 Jul 22;17(7):e0271575. doi: 10.1371/journal.pone.0271575 (PMC9307161; doi:10.1371/journal.pone.0271575)
Supplement: S2 Table — Note: H-Shannon-Wiener index, Dm-Mclntosh index, JP-Pielou index, Mc-Mclntosh index, S-Simpson index, Bp-Berger index, R = Patrick index, Me-Menhinick index (the same below). (PDF) [file pone.0271575.s004.pdf]

S2 Table Diversity index of different plant communities life-forms in the Aletai region

Desert community	life-forms	H	D_m	JP	Mc	S	Bp	R	Me
Seriphidium schrenkianum	Shrub	2.22	0.59	2.86	0.84	0.25	0.36	6	0.90
	Sub shrub	1.22	0.34	0.70	0.73	0.47	0.60	3	0.32
	Perennial herb	1.74	0.34	2.06	0.51	0.46	0.67	7	0.54
	Annual herb	2.18	0.50	2.40	0.67	0.32	0.52	8	1.05
Anabasis salsa	Shrub	1.98	0.48	2.55	0.75	0.30	0.41	6	0.52
	Sub shrub	0.43	0.07	0.90	0.17	0.86	0.93	3	0.24
	Perennial herb	1.9	0.51	2.72	0.79	0.31	0.4	5	0.70
	Annual herb	1.52	0.33	1.67	0.50	0.49	0.67	6	0.67
Artemisia arenaria	Shrub	1.58	0.52	3.32	0.99	0.33	0.36	3	0.60
	Sub shrub	1.26	0.35	0.63	0.75	0.47	0.60	3	0.28
	Perennial herb	1.76	0.45	0.95	0.73	0.36	0.50	5	0.59
	Annual herb	2.58	0.65	1.52	0.9	0.19	0.32	7	0.98
Haloxylon ammodendron	Shrub	1.42	0.44	2.98	0.87	0.40	0.49	3	0.48
	Sub shrub	1.98	0.60	3.29	0.98	0.26	0.32	4	0.75
	Perennial herb	1.82	0.53	2.60	0.72	0.36	0.53	5	1.21
	Annual herb	2.30	0.62	2.73	0.81	0.24	0.35	7	1.32
