# Supplementary material for: Factors driving the biomass and species richness of desert plants in northern Xinjiang China
Source: PLoS One. 2022 Jul 22;17(7):e0271575. doi: 10.1371/journal.pone.0271575 (PMC9307161; doi:10.1371/journal.pone.0271575)
Supplement: S3 Table — (PDF) [file pone.0271575.s005.pdf]

S3 Table Diversity index of different life-forms in the Yili region

Desert community	life-forms	H	D_m	JP	Mc	S	Bp	R	Me
Seriphidium borotalense	Shrub	1.86	0.47	0.83	0.76	0.33	0.51	5	0.61
	Sub shrub	1.75	0.49	2.46	0.68	0.29	0.48	4	1.25
	Perennial herb	1.98	0.49	0.75	0.72	0.30	0.39	7	0.66
	Annual herb	3.15	0.65	0.87	0.84	0.15	0.32	13	1.05
Ephedra sinica	Shrub	1.88	0.55	0.94	0.92	0.29	0.38	4	0.64
	Perennial herb	0.92	0.40	0.65	0.87	0.55	0.67	2	1.15
	Annual herb	2.33	0.57	0.82	0.76	0.26	0.41	8	0.92
Kochia prostrata	Shrub	1.91	0.47	2.62	0.68	0.33	0.30	7	0.68
	Sub shrub	1.55	0.54	3.24	0.97	0.35	0.41	3	0.72
	Perennial herb	1.74	0.62	1.93	0.75	0.26	0.39	8	1.67
	Annual herb	2.22	0.69	2.22	0.59	0.36	0.57	10	1.02
Haloxylon ammodendron	Shrub	1.96	0.52	2.52	0.69	0.35	0.55	6	1.28
	Perennial herb	1.68	0.46	2.40	0.68	0.40	0.54	5	0.86
	Annual herb	2.89	0.71	3.04	0.93	0.15	0.20	9	0.90
C.latens	Shrub	1.45	0.42	3.04	0.88	0.39	0.51	3	0.37
	Perennial herb	1.94	1.21	3.22	1.69	0.27	0.36	4	1.42

Desert community	life-forms	H	D_m	JP	Mc	S	Bp	R	Me
(continued)	Annual herb	2.69	0.54	2.42	0.73	0.22	0.39	13	1.16
Conyza canadensis	Shrub	1.46	0.35	2.42	0.64	0.46	0.64	4	0.35
	Perennial herb	2.39	0.62	3.07	0.88	0.23	0.33	6	0.96
	Annual herb	2.43	0.54	2.43	0.71	0.26	0.43	10	0.93
Stipa grandis	Shrub	1.45	0.34	1.87	0.55	0.45	0.62	6	0.27
	Sub shrub	1.71	0.49	2.45	0.71	0.37	0.50	5	1.02
	Perennial herb	2.31	0.61	2.73	0.81	0.24	0.33	7	1.16
	Annual herb	2.22	0.43	1.65	0.56	0.36	0.57	13	0.76
