# Supplementary material for: Factors driving the biomass and species richness of desert plants in northern Xinjiang China
Source: PLoS One. 2022 Jul 22;17(7):e0271575. doi: 10.1371/journal.pone.0271575 (PMC9307161; doi:10.1371/journal.pone.0271575)
Supplement: S4 Table — (PDF) [file pone.0271575.s006.pdf]

S4 Table Diversity index of different plant communities life-forms in the Bortala region

Desert community	life-forms	H	D_m	JP	Mc	S	Bp	R	Me
Ceratocarpus arenarius	Shrub	1.26	0.40	2.65	0.78	0.45	0.53	3	0.53
	Perennial herb	1.24	0.28	1.77	0.45	0.56	0.73	5	0.54
	Annual herb	3.33	0.71	3.08	0.92	0.12	0.24	12	1.07
Reaummuria soongorica	Shrub	1.53	0.52	3.20	0.94	0.36	0.47	3	0.69
	Perennial herb	0.76	0.18	1.60	0.36	0.72	0.84	3	0.60
Haloxylon ammodendron	Annual herb	2.06	0.49	2.29	0.68	0.31	0.45	8	1.09
	Shrub	2.14	0.54	2.53	0.76	0.28	0.40	7	0.85
	Perennial herb	2.19	0.49	2.19	0.62	0.33	0.53	10	1.25
	Annual herb	3.20	0.66	2.54	0.81	0.15	0.27	18	1.10
Nanophyton erinaceum	Shrub	1.16	0.31	2.43	0.62	0.54	0.71	3	0.42
	Perennial herb	2.26	0.71	3.23	0.96	0.22	0.31	5	1.25
	Annual herb	2.34	0.58	2.77	0.83	0.23	0.33	7	0.86
