# Supplementary material for: Factors driving the biomass and species richness of desert plants in northern Xinjiang China
Source: PLoS One. 2022 Jul 22;17(7):e0271575. doi: 10.1371/journal.pone.0271575 (PMC9307161; doi:10.1371/journal.pone.0271575)
Supplement: S5 Table — (PDF) [file pone.0271575.s007.pdf]

S5 Table Diversity index of different plant communities life-forms in the east desert subregion of the eastern Tianshan Moutain of Xinjiang

Desert community	life-forms	H	D_m	JP	Mc	S	Bp	R	Me
Iljinia regelii	Shrub	1.21	0.34	2.52	0.72	0.48	0.50	3	0.34
	Perennial herb	1.50	0.78	3.15	0.92	0.38	0.45	3	0.50
	Annual herb	0.49	0.14	1.65	0.31	0.82	1.02	2	0.63
Ephedra przewalskii	Shrub	0.92	0.31	3.05	0.87	0.55	0.66	2	0.38
	Sub Shrub	1.24	0.42	2.52	0.77	0.46	0.51	3	0.69
	Perennial herb	0.95	0.40	3.31	1.02	0.50	0.53	2	0.55
	Annual herb	0.94	0.38	3.32	0.99	0.50	0.48	2	0.46
Halostachys caspica	Shrub	1.20	0.41	1.42	0.68	0.33	0.45	5	0.97
	Annual herb	1.24	0.44	1.44	0.72	0.36	0.42	6	1.12
