# Supplementary material for: Factors driving the biomass and species richness of desert plants in northern Xinjiang China
Source: PLoS One. 2022 Jul 22;17(7):e0271575. doi: 10.1371/journal.pone.0271575 (PMC9307161; doi:10.1371/journal.pone.0271575)
Supplement: S6 Table — (PDF) [file pone.0271575.s008.pdf]

**S6 Table** Diversity index of different plant communities life-forms in the Tacheng region

| Desert community | life-forms | H | $D_m$ | JP | Mc | S | Bp | R | Me |
|---|---|---|---|---|---|---|---|---|---|
| *Seriphidium schrenkianum* | Shrub | 0.93 | 0.17 | 1.19 | 0.26 | 0.72 | 0.84 | 6 | 0.58 |
| | Sub shrub | 1.02 | 0.25 | 2.06 | 0.50 | 0.62 | 0.77 | 3 | 0.43 |
| | Perennial herb | 1.47 | 0.35 | 2.43 | 0.66 | 0.45 | 0.62 | 4 | 0.30 |
| | Annual herb | 3.02 | 0.65 | 2.51 | 0.74 | 0.20 | 0.40 | 16 | 1.02 |
| *Kochiaprostrata* | Shrub | 1.52 | 0.48 | 3.18 | 0.94 | 0.36 | 0.43 | 3 | 0.55 |
| | Sub shrub | 1.42 | 0.38 | 2.37 | 0.65 | 0.45 | 0.63 | 4 | 0.58 |
| | Perennial herb | 2.05 | 0.61 | 2.86 | 0.86 | 0.28 | 0.30 | 5 | 1.12 |
| | Annual herb | 2.72 | 0.63 | 2.71 | 0.82 | 0.19 | 0.34 | 10 | 1.08 |
| *Reaumuria soongorica* | Shrub | 2.21 | 0.57 | 2.62 | 0.78 | 0.26 | 0.33 | 7 | 0.95 |
| | Sub shrub | 0.99 | 0.49 | 3.30 | 0.95 | 0.52 | 0.60 | 2 | 0.92 |
| | Perennial herb | 1.34 | 0.32 | 2.22 | 0.53 | 0.52 | 0.70 | 4 | 0.45 |
| | Annual herb | 2.61 | 0.60 | 2.60 | 0.80 | 0.21 | 0.32 | 10 | 0.62 |
| *Nanophyton erinaceum* | Shrub | 1.31 | 0.33 | 2.17 | 0.58 | 0.50 | 0.66 | 4 | 0.46 |
| | Sub shrub | 1.38 | 0.47 | 2.89 | 0.84 | 0.41 | 0.50 | 3 | 0.70 |
| | Perennial herb | 0.99 | 0.37 | 3.28 | 1.05 | 0.51 | 0.57 | 2 | 0.43 |
| | Annual herb | 3.05 | 0.65 | 2.59 | 0.80 | 0.16 | 0.27 | 15 | 1.26 |
