# Supplementary material for: Factors driving the biomass and species richness of desert plants in northern Xinjiang China
Source: PLoS One. 2022 Jul 22;17(7):e0271575. doi: 10.1371/journal.pone.0271575 (PMC9307161; doi:10.1371/journal.pone.0271575)
Supplement: S7 Table — (PDF) [file pone.0271575.s009.pdf]

S7 Table Diversity index of different plant communities life-forms in the desert subregion of Changji-Urumqi region

Desert community	life-forms	H	D_m	JP	Mc	S	Bp	R	Me
Petrosimonia sibirica	Shrub	1.54	0.49	1.04	0.96	0.35	0.43	3	0.55
	Sub shrub	0.54	0.17	0.60	0.35	0.78	0.88	2	0.71
	Perennial herb	2.87	0.81	2.28	0.95	0.15	0.22	8	1.89
	Annual herb	2.35	0.47	1.09	0.54	0.33	0.53	14	1.16
Seriphidium schrenkianum	Shrub	0.21	0.12	0.15	0.23	0.77	0.88	4	0.34
	Sub shrub	1.25	0.35	0.77	0.75	0.50	0.66	3	0.47
	Perennial herb	2.79	0.68	1.67	0.89	0.17	0.25	9	1.31
	Annual herb	2.48	0.55	1.24	0.78	0.26	0.41	12	1.20
Reaumuria soongorica	Shrub	1.11	0.33	2.32	0.66	0.51	0.64	3	0.57
	Sub shrub	1.90	0.65	3.16	0.95	0.28	0.32	4	0.80
	Perennial herb	2.13	1.55	2.72	0.76	0.30	0.44	6	1.73
	Annual herb	2.02	0.42	2.56	0.74	0.32	0.42	6	0.64
Haloxylon ammodendron	Shrub	1.05	0.34	0.63	1.15	0.50	0.51	2	0.32
	Sub shrub	1.87	0.56	1.29	0.91	0.26	0.32	4	0.75
	Perennial herb	2.62	0.64	1.59	0.75	0.15	0.33	10	1.50
	Annual herb	2.86	0.68	1.43	0.95	0.24	0.19	8	0.80
	Shrub	2.15	0.61	3.28	0.98	0.26	0.32	4	0.76

Ceratocarpus utriculosus	Sub Shrub	1.58	0.52	3.31	0.99	0.33	0.31	3	0.58
Desert community	life-forms	H	D_m	JP	Mc	S	Bp	R	Me
(continued)	Perennial herb	1.55	0.35	2.57	0.67	0.44	0.62	4	0.51
	Annual herb	2.75	0.65	2.99	0.89	0.18	0.31	8	1.13
Nanophyton erinaceum	Shrub	0.54	0.13	1.80	0.39	0.78	0.88	2	0.28
	Annual herb	1.48	0.42	2.47	0.74	0.40	0.45	4	0.50
